# Diagnosing Rickets in Early Modern England: Statistical Evidence and Social Response

**DOI:** 10.1093/shm/hkab019

**Published:** 2021-10-05

**Authors:** Gill Newton

**Affiliations:** CAMPOP, Department of Geography, University of Cambridge, Downing Place, Cambridge, CB2 3EN, UK

**Keywords:** rickets, child mortality, air pollution, infant feeding, cause of death reporting

## Abstract

Seventeenth-century UK experienced an epidemic of the newly recognised disease rickets, its nutritional and environmental causes then unknown. This is evident from parish burial registers, the London Bills of Mortality, and contemporary medical descriptions and treatments. Rickets appeared to be killing 2–8 per cent of urbanites, especially wealthy children.

Rickets emerged as a threat to child health in early modern UK as a result of coal dependency and climate, and social differences in infant and child feeding. Physicians investigating rickets showed concern for rich children’s diets. Lack of breastfeeding promoted calcium deficiency among wealthy infants, while poorer children’s meagre childhood diet retarded recovery.

The seasonality and age incidence of rickets deaths corroborate this diagnosis, but after 1700 rickets deaths dwindled even as medical treatises and osteological evidence suggest rickets morbidity increased. Chronology and share of mortality of other causes relating to rickets morbidity are considered: scurvy, hydrocephalus and whooping cough.

Diagnosis from cause of death descriptors is a central problem of historical investigations of mortality, especially those covering a period of several centuries.^1^ Names given to diseases may change over time, and assessing how far causes of death represent single, consistently identified diseases as we know them today is a complex proposition.

Statistical investigations of recorded causes of death provide clues gleaned from seasonality and age incidence. Direct evidence of disease may be gained from archaeological investigation of human remains. Treatises and other publications by medical practitioners provide invaluable contemporary testimonies of patients’ symptoms, although medical practitioners were not involved in compiling seventeenth- and eighteenth-century mortality records, and most showed little interest in them.^2^

In seventeenth-century UK, the environmental and nutritional deficiency disease rickets emerged as an apparently new disease of childhood. To contemporaries, it was fascinating because of this newness, and alarming because of its rapid rise in incidence, its apparently disproportionately mortal effect on children from wealthy households, and the deformities and debility that it caused. The aim of this article is twofold. Firstly, to demonstrate the value of pursuing plural lines of enquiry to diagnose historical causes of death, through the exemplar of rickets. Secondly, to use this exploration of a deficiency disease to probe environmental circumstances and nutritional status of seventeenth- and eighteenth-century urbanites, especially children, at a time when overall English child mortality rates were severe, and worsening most markedly of all in London.^3^

## Rickets and the urban environment

Rickets is perhaps best known historically as a disease of childhood in the urban slum, but it is still seen today, even in affluent countries, among children who have received restricted exposure to sunlight, an inadequate diet, or both, and are therefore deficient in Vitamin D and/or calcium. Rickets has been known in medicine as the ‘English disease’ since its first description by Whistler in 1645 and is still referred to as such.^4^ Its status as a recurrent public health concern in England is largely in consequence of seasonal and climatic deficiencies in sunlight. New public health guidance in 2016 recommended a daily Vitamin D supplement for all children under 4 years and seasonal supplements for adults, in recognition of the paucity of dietary sources and the difficulty of obtaining sufficient Vitamin D all year round.^5^

Vitamin D is a key part of calcium metabolism, which when disrupted impairs bone mineralisation and growth in children, leading to the well-known and visually distinctive skeletal deformities of rickets, affecting especially the long bones, ribs, skull and pelvis. Pregnant women, their foetuses and young children are particularly susceptible because of the high mineral requirements of growing bone. In older children, difficulties in standing and walking are typical. In infants, acute hypocalcaemia can be fatal. However, if calcium intake is high, and bioavailability good because the food contains soluble calcium salts that are well-suited to human digestion (as with breast-milk), passive absorption of calcium without Vitamin D can occur.^6^

Natural dietary sources of Vitamin D being few and meagre, the primacy of sunlight in providing Vitamin D has drawn some scholarly attention to the relationship between rickets and environmental conditions in England during industrialisation. For example, Hollick notes that in 1822, the Polish physician Sniadecki referred to rickets as ‘the English disease’ when making his ground-breaking observation that sufficient exposure to sunlight could cure urban children of rickets.^7^ Hardy discusses a British Medical Association report from 1889 indicating geographical prevalence of rickets morbidity in Britain, which attested to the association of rickets with urban coal use.^8^ Where present in rural places, it was shown to be more common in the south than in the north.

Increasing fogs and gloom from coal smoke in Victorian and Edwardian London are well-documented.^9^ However, urban coal dependency in Britain began much earlier than the nineteenth century. Since before Elizabethan times, London had been burning mineral coal as a fuel, brought by sea predominantly from mining areas in northern England near Newcastle. At first, concomitant environmental nuisance was localised, from industrial furnaces. Brimblecombe has drawn attention to the emergence of rickets in seventeenth century London as domestic coal use rocketed and air pollution increased across the whole city.^10^ London smogs of the 1650s whose impact on available sunlight made it impossible for plants to photosynthesise well enough to bear fruit were vividly described by John Evelyn in *Fumifugium*, which also compares London unfavourably to elsewhere: ‘For when in all other places the Aer is most Serene and Pure, it is here Ecclipsed with such a Cloud of Sulphure, as the Sun it self, which gives day to all the World besides, is hardly able to penetrate and impart it here.’ Blockades on coal leaving Newcastle during the Civil War years provided a natural experiment that seemed to prove coal was the culprit.^11^ He and other astute observers, all members of the influential Royal Society, were keenly aware of a change in London’s air quality by the mid-seventeenth century. Evelyn, John Graunt, and later the economic statistician Gregory King, all suspected ‘sea coal’ smoke had reached such proportions as to be having a deleterious effect on the health of citizens, with several linking it to asthma and another supposedly ‘English disease’ of the lungs: consumption.^12^ Well-travelled diplomat and natural philosopher Kenelm Digby judged that because of coal smoke the air was worse in London than in other European cities like Liège or Paris.^13^

Rising coal imports to London in the sixteenth century attest to the fast-growing adoption of coal as a fuel.^14^ London was becoming coal dependent, as were other seventeenth-century British towns including Oxford, Cambridge and its second city, Dublin. Cold winters added to fuel demands for heating. More River Thames freeze-overs in London are documented between 1650 and 1699 than in the whole of the eighteenth century or the preceding one hundred years since 1550, and in the continuous record from 1659 to the present provided by the Central England Temperature series, seventeenth-century winter temperatures are the lowest.^15^

Importantly for the geographical specificity of rickets, and perhaps other so-called ‘English’ diseases, this degree of dependence on coal as a fuel at such an early date was a peculiarly English phenomenon. Elsewhere in Europe there were densely populated cities consuming large amounts of fuel, particularly in the Low Countries, but the fuel there was predominantly peat, and only selectively in certain industries were smoky ‘sea-coals’ used.^16^ Elsewhere wood remained almost the only fuel. Paris, for instance, remained entirely dependent on wood and charcoal for fuel until well after 1800.^17^ The rapidity of London’s population and industrial growth could only be sustained by coal use, given the relative paucity of accessible woodland or peat. London wood prices rose fast to outstrip coal prices and wage increases in the first half of the seventeenth century.^18^ There were only short periods during the whole of the seventeenth century in which disruption to coal supply and shipping made coal the more expensive fuel, as during the Civil War, which also hastened the demise of woodland in the vicinity of London.^19^

Intensification of the built environment in poorer London suburbs over the seventeenth century probably exacerbated reduced exposure to sunlight from smog.^20^ Infill development behind the main streets increased population density and overcrowding, especially in the densely packed narrow alleys and courts where the cheaper rentals were.^21^ The timbered housing stock that seventeenth-century London still possessed in abundance, with upper stories stepped outwards from the building’s footprint to overhang the street, contributed to the gloom. Little of the old city suburbs were burnt in the Great Fire in 1666 so there was no compulsion to rebuild them. The post-Fire rebuilding of the city centre and eighteenth-century expansion of Westminster, by contrast, involved straight-sided brick houses intended to prevent the spread of fire, a side benefit of which was to leave more open and unshaded space between these buildings inhabited by the wealthy.

## The epidemiological evidence

The seventeenth-century rickets epidemic is observable through annual rickets deaths in the seventeenth-century London Bills of Mortality, compiled from the reports of clerks who also kept the burial register in each parish. Individual rickets burials can also be observed at source, among causes of deaths that were recorded in the burial registers of some parishes.

**Fig. 1.**
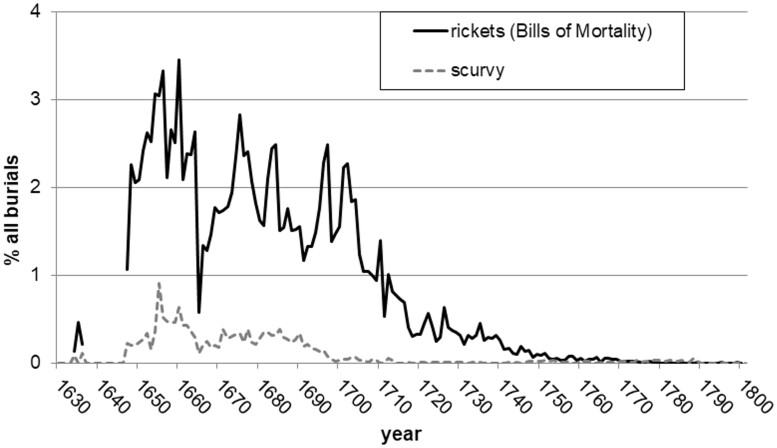
Proportion of all London deaths attributed to rickets and scurvy, 1630–1800 Source: data taken from John Marshall, *Mortality of the Metropolis […]* (London, printed by J. Haddon for J. Marshall, 1832).

The number of rickets deaths in London increased rapidly after its first appearance in the Annual Bill of Mortality for 1634, when 14 died of rickets. Scattered weekly Bills of Mortality attest to the presence of rickets in every year of incomplete annual data between 1635 and 1647, ending with 142 rickets deaths in the near-complete 48 week series of 1647. By the 1650s 300–400 rickets deaths were usual in each year. Rickets sustained a similar number of deaths following the population changes and temporary disruption to record-keeping of the Great Plague of 1665 and Great Fire of 1666. As shown in Figure 1, at its peak, rickets accounted for 3 per cent of all London burials. Yet after 1700 rickets declined steeply as a cause of death, dwindling to just 20 deaths per year by 1750. There are no rickets burials in London after 1811. Nor did rickets remain a cause of death elsewhere. Despite the disease’s later association with industrial towns, among hundreds of thousands of burials taken from parish registers recording causes of deaths in 1770–1812 drawn from Manchester, Leeds, York and Liverpool and other burgeoning towns, only one rickets fatality has been found in these locations, in Wigan in 1807.^22^ The only other exception found to the late eighteenth century disappearance of rickets is in the physician John Haygarth’s Bills of Mortality for Chester, where seven children died of rickets in 1772.^23^

The rise and fall in rickets as a cause of death raises several questions. Firstly, why did rickets emerge as a cause of death in the seventeenth century? Secondly, why did its death toll apparently dwindle to zero in the eighteenth century although, as we shall see, there is every indication that the disease remained prevalent? And thirdly, did rickets actually cause the deaths of those ascribed to the disease?

**Fig. 2.**
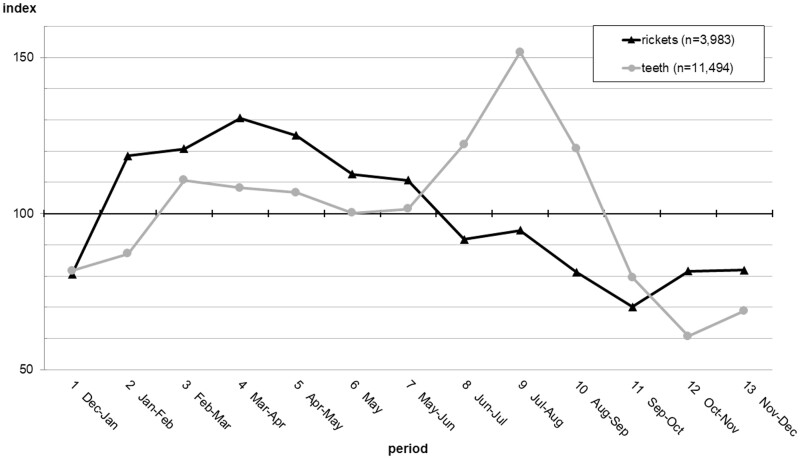
Seasonality of rickets burials and teeth burials from the weekly London Bills of Mortality between 1670 and 1679 Source: Richard Smith, Romola Davenport and Gill Newton, *London weekly bills of mortality, 1644–1849.* [data collection]. 2020, UK Data Service, SN: 854104, http://doi.org/10.5255/UKDA-SN-854104 (last accessed February 2021).

An examination of rickets seasonality from the weekly Bills of Mortality is useful to establish at the outset whether rickets had any cohesive identity. Only partial years survive before 1665, and the second half of the 1660s are disrupted by plague, fire and subsequent reconstruction. Therefore the 3,983 rickets burials from the following decade 1670–9 were analysed. Their seasonality in thirteen divisions of the year is shown in Figure 2.^24^ Approximate calendar months each period represents are given below the *x*-axis for reference. Deaths are shown relative to an index of 100, which would indicate an equal share of burials in each division of the year. Teeth (teething) is a major cause of death applicable to children in roughly the same young age group as rickets deaths are likely to fall, so the index values for teeth burials in the same decade are also plotted for comparison.

The seasonality of rickets and teeth deaths are strikingly distinct from each other, with rickets deaths peaking in late winter and spring, in contrast to the strong summer peak of teeth burials (such a peak is often associated with increased transmission of diarrhoeal disease in the warmer months). The seasonality of rickets deaths is certainly not random, and accords with the seasonal incidence of Vitamin D deficiency expected today at high latitudes, where the late spring months of April and May follow many months of total deprivation and Vitamin D deficiency is at its most acute. The timing of the initial rise in the rickets seasonal index in period 12 (October–November) is coincident with the beginning of the period when Vitamin D cannot be synthesised.

Other essential micronutrients such as Vitamin C, found in fresh produce, would also have been less available in the winter months outside the growing season, and it is possible that some of the seventeenth-century rickets sufferers also had other deficiency diseases, such as scurvy. The chronology of scurvy as a cause of death descriptor in the Bills of Mortality, also shown in Figure 1, is similar to that of rickets. Its seasonality peaks strongly in winter at index value 152 in period 2, for 1,629 scurvy burials 1670–1719. Admittedly scurvy is a particularly ambiguous cause of death descriptor in the early modern period that could be conflated with venereal diseases. It also killed adults, whereas no adult rickets deaths have been found.^25^ However, Glisson, the respected seventeenth-century medical authority still credited with the ‘discovery’ of rickets, noted that scurvy sometimes ‘conjoyned’ rickets in infants.^26^ In the nineteenth century, comorbidity of rickets and scurvy was well-documented in Cheadle and Barlow’s ‘scurvy-rickets’ cases at Great Ormond Street Hospital in the 1870s and 1880s, attributed in part to nutritionally inappropriate new proprietary infant feeding products then popular in richer households. ^27^ Seventeenth-century medical suspicions of a causal connection between infant feeding and rickets will be discussed in the section of this article entitled ‘Medical response: inspecting the dead and treating the living’.

The pattern of few summer and early autumn rickets deaths compared to the spring and winter death toll that is apparent for London as a whole is also discernible in some of its constituent parishes that recorded causes of death, providing an important check on the consistency of identification of disease and also allowing some examination of earlier rickets deaths from the 1650s. The seasonality of rickets burials in the populous northwestern suburb of St Giles Cripplegate in the 1650s is tipped towards the spring and winter months, with fewer deaths in summer and autumn. In three complete years 1654–56 covered by the burial register, 39 rickets burials fell between December and May and only 15 between June and November. In the small central city parish of St Michael Cornhill also in the early 1650s, during four complete years 1653–57, 8 of 11 rickets burials occurred between December and May.

Parish registers are particularly valuable for the individual-level glimpses of what sorts of Londoners rickets affected they permit. Among causes of death ascribed to children, there is also local variation in the proportion said to die of rickets. Most usefully of all, parish register sampling permits scrutiny of age incidence of rickets in different parts of the metropolis. The Bills of Mortality tell us nothing about the ages of those suffering from rickets, as age breakdowns were not included until 1728, and then not on a cause-by-cause basis.

The highest rate of rickets burials in parish registers was discovered in the small parish of St Michael Cornhill in the city centre, where rickets represents the joint second ranking cause of death, together with consumption, with only convulsions claiming more lives. Few parish registers record cause of death at this time, but the importance of the St Michael Cornhill rickets evidence must be tempered with cautious interpretation, since it is a small parish and any newly emergent disease might be identified idiosyncratically by some parish officers. Nonetheless, the existence of seasonal and, as we shall see, age patterns in these and other parish data are suggestive. In combination with contemporary medical treatises and osteological evidence discussed below, they bolster the case for social status-related differences in rickets prevalence.

The 11 rickets deaths occurring in St Michael Cornhill occurred during a 4 year period when causes of death are almost universally recorded (for 140 of 143 burials in total).^28^ In this wealthy parish rickets deaths constituted 7.7 per cent of all burials, more than triple the all-London proportion at that time. The register does not state ages, but they are calculable from record linkage to baptisms, some of which include the date of birth. In this way, the ages of 10 of the 11 rickets burials were recovered. All were very young, aged between 1 and 22 months, and the mean age was 10 completed months.^29^

The occupations of adult men dying in the parish give some indication of the social range to be found in St Michael Cornhill. Among 34 men buried in these four years, one in five was an upholder (the London guild of upholsterers). High status occupations such as gentlemen, doctor, linen draper and merchant were well-represented, but lower status occupations such as weaver and porter were also present. Further evidence on status can be gained from the 1662 Hearth Tax for Cornhill Ward. Five of the fathers of rickets casualties appear as householders in this assessment. In the entire ward, the median number of hearths assessed was 4. Two of the fathers who appear as householders were assessed for above average dwellings of 9 and 13 hearths, the latter’s high social standing indicated by the title of esquire. One father was assessed for 4 hearths, the median; and two fathers for slightly below average dwellings of 3 hearths.^30^ Thus, the status of traceable fathers of rickets burials as indicated by the size of their dwellings, was at or slightly above average for wealthy Cornhill Ward.

In the more populous suburbs, in St Martin-in-the-Fields, the Sextons Burial Books extant for 1685–87 and 1694–1703 record rickets as the cause of death for 331 persons among 8,718 child burials, or 15,856 burials in total.^31^ Thus rickets in this Westminster parish accounts for 3.9 per cent of all child burials in the parish, and 2.1 per cent of all burials, very similar to the all-London rate.^32^ In the less distant suburban parish of Cripplegate, just beyond the city wall, the 75 rickets burials between 1653 and 1657 formed a similar proportion of 1.9 per cent of total burials, although by the 1680s this had dropped to 1.3 per cent of total burials and there were only 7 rickets deaths in over 11,500 burials by the 1690s.

The Cripplegate registers do not state the age of the deceased, so ages at death were again calculated by linking burial records to baptism records. Record linkage in this parish is more challenging because of the much larger number of records, with 4,000 burials registered in the same 4-year period as the 143 registered in St Michael Cornhill. This gives rise to ambiguities from common names. A sub-sample of 25 of the 75 rickets burials in these four years from the 1650s could be assigned precise ages through an unambiguous match with a baptism record.^33^ The mean age of this sample was 21 completed months, more than twice the mean age in Cornhill. 17 of the 25 Cripplegate children were aged between 12 and 36 months. Only one-fifth were aged under 1 year, although the sample did include children as young as 2 or 3 months. The oldest child was 60 months, or 5 years.

Since these are a minority of total rickets fatalities in Cripplegate, the question of representativeness arises. However, if there is bias in the ages, it should be in a direction that suppresses the true extent of the difference with the Cornhill ages at burial, since successful record linkage is likely to over-represent the children who died at the youngest ages before their parents could move elsewhere. In consequence, the true average age of all rickets burials in Cripplegate was probably somewhat older than the linked sample suggests. The children of the only two high status fathers in the Cripplegate sample, a gentleman and a lawyer, both died very young at under 1 year of age. Fathers who were a printer and stationer, and a soapmaker might have had some stock or equipment of value, depending on the size of their businesses. Other than these, fathers’ occupations fell in Brodsky Eliot’s fourth and lowest status group of seventeenth-century London occupations. ^34^ Most numerous among these low status craftsmen were weavers, tailors and glovers, accounting for 11 fathers.

The incidence of rickets mortality in some city centre parishes more closely resembled the lower rate prevailing in the suburbs. In St Helen Bishopsgate, of 186 burials between September 1653 and April 1659 only four were rickets deaths, about 2 per cent of the total. Three of them had high status fathers: a gentleman, a perfumer, a merchant; the fourth was the child of a locksmith, a skilled trade.^35^ Two can be traced to their baptism record and were aged 9 months and 20 months old when buried. This, incidentally, was the parish where the physician Whistler, who wrote the first medical treatise on rickets, lived from July 1654 onwards. Glisson’s parish of residence, St Bride Fleet Street, records no causes of death in its registers, whereas St Michael Cornhill, with its high proportion of rickets burials, was home to John Graunt. Other city centre parishes recorded causes of death but attributed none to rickets, although their typically small size means this could have occurred by chance. In St Stephen Coleman Street, for example, causes of death are recorded continuously for 17 months between 1655/6 and 1657, but there is no mention of rickets among 133 burials in total.^36^

In the poor but populous suburban parish of St John Wapping in the East End, rickets as a cause of death descriptor seems to have passed in and out of use, suggesting that while rickets was known in this part of the city, not all Searchers regarded it as a legitimate cause of death. Extant cause of death recording begins in Wapping in June 1665, where one rickets burial is listed within the first 10 entries.^37^ Registration lapses soon thereafter, then resumes in September 1666 just after the Great Fire (which did not affect Wapping directly) and carries on unbroken for decades. But there are no rickets burials for the first 6 years and more, so that over 800 burials pass by attributed to other causes before, in June 1673, the first recorded rickets burial subsequent to the one of 1665 occurs. More swiftly follow, bringing the share of Wapping burials ascribed to rickets in the 1670s to 1.5 per cent, but this declines to 0.9 per cent by the end of the seventeenth century. In this poor riverside parish rickets burials occurred at ages similar to those in Cripplegate, and older than in wealthy Cornhill. Among 6 individuals linked to a baptism record out of 18 rickets burials between 1673 and 1680, the mean age was 21 completed months, the youngest 4 months and the oldest 39 months. Ten of the 18 rickets burials were marked poor, meaning that their fathers were unable to afford a funeral and the cost of burial was absorbed by the parish. Unfortunately none of this large pauper proportion could be traced to a baptism record, so their ages remain unknown. Of the six who were traceable, the three with stated occupations were a mariner, a mealman and a ship chandler. The first of these is a low status occupation; the other two might entail some holdings of stock and therefore financial reserves.

In mid-eighteenth-century Whitechapel, another East End parish adjacent to Wapping, there was a similarly large proportion of pauper burials. 20 of 38 rickets burials recorded between 1743 and 1768 were marked poor. Here the stated ages of virtually all children (>99 per cent) are given, so it is possible to check whether poor or non-poor status has any effect on the age at burial of rickets fatalities. The median age among both pauper and non-pauper rickets burials was 2 years, the oldest was 5 years, and none were under 1 year. The lack of known ages for paupers among the Wapping burials thus should not distort the evidence, and the Wapping sample may be taken as typical with regard to age.^38^

**Fig. 3.**
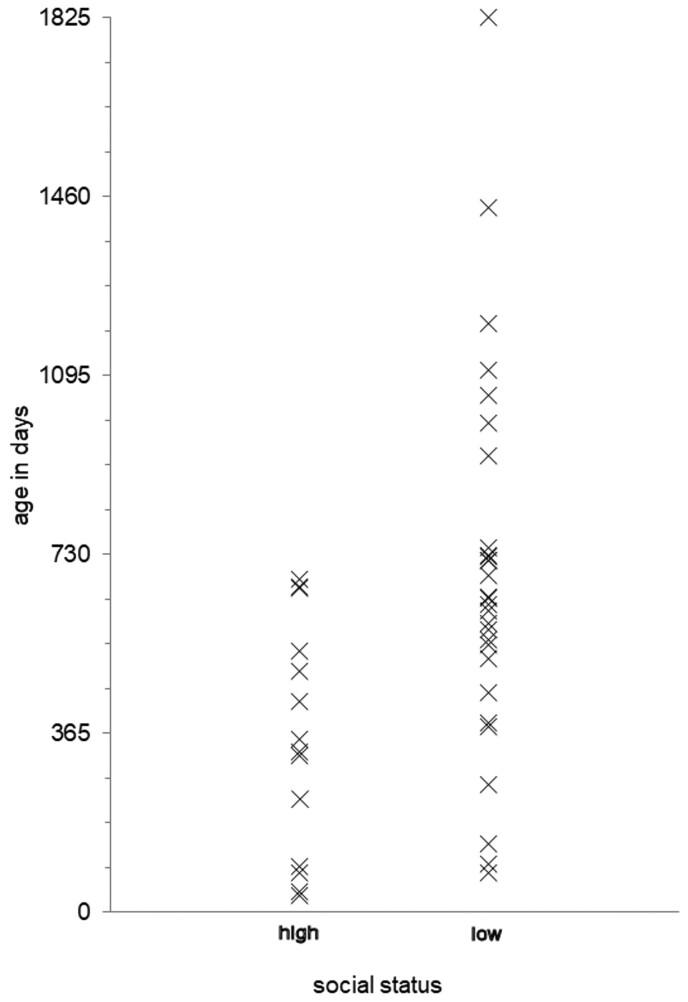
Ages of 14 high-status and 29 low-status children dying of rickets 1653–1680 Source: Record linkage from baptisms and burials recorded in the parish registers of St Michael Cornhill, St Helen Bishopsgate, St Giles Cripplegate and St John Wapping.

In Figure 3, the 43 seventeenth-century burials of children whose ages can be calculated as discussed above have been divided into two sample groups representing high and low socioeconomic status, according to their father’s occupation and parish of residence. The difference between the two groups is immediately apparent. The deaths of the high-status children are concentrated at younger ages than the low status children. All high-status deaths occurred at ages under 2 years and most were under 12 months, whereas low status children’s deaths from rickets extended to older ages of up to five years, and were concentrated in the 18–24 month age group. A *t*-test conducted to compare the two samples confirmed that the difference between them is statistically significant, at the 1% level (*p *=* *0.0045, two-tailed).

Taking the ages of rickets deaths as an indicator of the ages at which a child could be visibly suffering from the disease, Figure 3 suggests that high status children who survived past their second birthday recovered more swiftly than their low status counterparts. That does not imply that rich children had an overall mortality advantage. On the contrary, mortality levels in late seventeenth-century London were high, and the infectious diseases that killed most children did not spare the rich, for both the infant mortality rate and the early childhood mortality rate of children aged 1–4 years frequently exceeded 200 per thousand in rich and poor parishes, and among socially heterogeneous groups like the Quakers.^39^ Also, rickets neonatal deaths seem to have most afflicted the rich.

Country nursing of children potentially affects the observable ages at which urban children of different social statuses die. However, the proportion of London’s mercantile elite sending their children away to nurse was already declining by the first half of the seventeenth century, while the high status children here observed dying of rickets in their home parishes are spread throughout the first two years of life, the age group found most likely to be absent and probably away at nurse in 1695.^40^ Still, gentry families in particular continued this custom, and in fashionable Westminster it has been suggested that nearly one third of children were away at nurse in the mid-eighteenth century. ^41^ For such children, relief from smog might have aided recovery from rickets, if given a suitable diet.

Wealth differentials in the propensity of children to suffer from rickets at different ages are also evident from archaeological excavations. Both active and healed instances of rickets have been identified in English skeletal remains from the early modern period, and the largest studies come from London burial grounds.^42^ A central difficulty with osteological evidence is that assemblages often span very long periods with only a few specimens more precisely dated. But there are exceptions, as when monumental plaques or coffin plates yield shorter burial usage periods or exact dates of death. Such was the case in the Spitalfields project, which involved exhumation of high status burials from the crypts of an East London church frequented by prosperous Huguenot silkweavers and their descendants between 1729 and 1859.^43^ Low status burials from the nearby New Churchyard, on the site now known as Broadgate, also in East London, for the period 1569–1720, have also been examined for signs of rickets. ^44^ These two studies found 21 children under seven years of known age with rickets in total, including seven infants, although the bones of infants are usually under-represented in skeletal assemblages. Almost all of the high status Spitalfields children with signs of rickets were very young, aged under two years. In contrast, the ages of the seven Broadgate children with rickets were spread more evenly throughout early childhood, from infancy to the age of 5 years.

The osteological evidence supports the hypothesis that rich children suffered from rickets earlier in life and recovered sooner and more swiftly than poor children, and that the pattern persisted into the eighteenth century. However, in the less well preserved Broadgate specimens, identification of rickets relied heavily on the presence of bowing of the leg bones, which may over-represent older ages since although present in active cases, it is most obvious in children (and adults) with healed rickets where growth has continued.^45^ Still, no child older than 3 years in the Spitalfields sample was identified as having rickets, even though such cases ought to have been easier to recognise there too. The extreme concentration at young ages in Spitalfields further suggests real differences between the two populations.

The osteological evidence is particularly important because it provides almost the only quantifiable estimate of rickets prevalence in the eighteenth century, thus further proving the near-total disappearance of the disease after 1750 implied by the Bills of Mortality is implausible. Still, there may have been some amelioration in rickets prevalence among wealthy children in the eighteenth century, for the relative frequency of rickets among burials of poor young children was somewhat higher for the earlier pre-1720 period of Broadgate burials than among the eighteenth and nineteenth century rich children of Spitalfields, at 20 per cent of child burials aged under 7 years compared to 14.7 per cent in Spitalfields. None of the rickets evidence from Spitalfields is pre-1750, but if it did include earlier burials the situation might well be reversed, given the relative social status of the two groups, and the higher apparent prevalence of rickets among wealthy children in the seventeenth century. These substantial rates of rickets prevalence are well on the way to those observed in the 1850s and 1860s among hundreds of children admitted to London and Manchester clinics, which ranged from 20 per cent to 30.3 per cent.^46^

The disappearance of rickets as a cause of death raises the question of whether rickets ever did cause the deaths attributed to it, and to what alternative cause or causes eighteenth century children dying with the symptoms of rickets might have been assigned. Some of the rickets mortality may genuinely document acute, lethal cases of hypocalcaemia, but it seems likely that many of those said to die of rickets actually died of other causes, including infantile scurvy.

Importantly for the impact of rickets on child mortality in general, the combination of weakened thoracic muscles and soft rib bones it induces makes children with rickets more susceptible to respiratory illness, because the skeletal support to the lungs provided by the ribcage is impaired, as the Philadelphia clinician Parry later concluded from both observation and autopsy, remarking that ‘all know how these [rickety] children suffer from pulmonary symptoms’.^47^ In developing countries today, rickets is associated with greatly increased risk of respiratory infection. In one study, Ethiopian children with rickets had thirteen times higher incidence of pneumonia than other children, and in Jordan among young children retrospectively diagnosed with rickets, it was respiratory infections that actually lead to hospitalisation.^48^

**Fig. 4.**
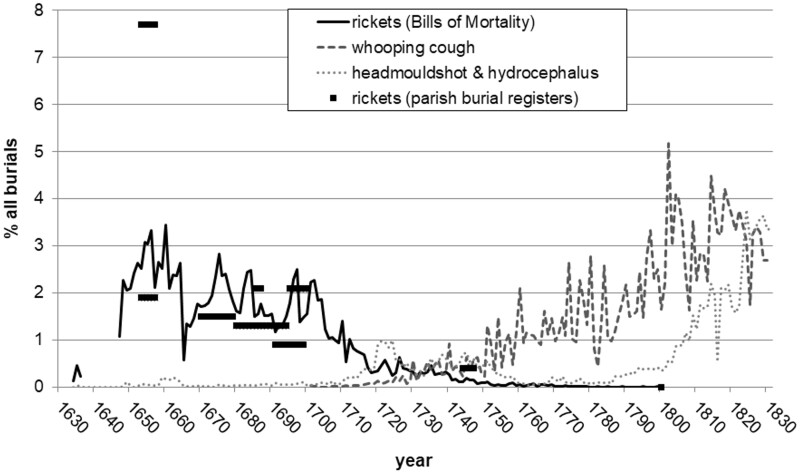
Rickets, whooping cough and head deformities in the London Bills of Mortality, 1630–1830 Source: John Marshall, *Mortality of the Metropolis*; parish burial registers of St Michael Cornhill, St Giles Cripplegate, St Martin in the Fields, St John Wapping and St Mary Whitechapel.

Hardy has conjectured that there was an association between nineteenth century rickets morbidity and mortality from a respiratory disease that had by then become a major cause of death in young children: whooping cough.^49^ In light of this, it is informative to consider the timing of the initial appearance and rise in whooping cough and ‘chincough’ fatalities recorded in the eighteenth-century Bills of Mortality. The use of chincough/whooping cough as a cause of death in the Bills begins around 1700, and it rises in prominence from the 1720s onwards, as shown in Figure 4.^50^ This rise is coincident with the tail end of the decline in deaths attributed to rickets deaths, and whooping cough deaths continued to rise rapidly thereafter, assuming by the end of the eighteenth century a similar share of overall mortality as taken by rickets at the height of its epidemic.

Besides whooping cough, there are two further causes of death which may be partially indicative of rickets morbidity, since deformations of the head was the most widely recognised symptom. These are horseshoe head and head-mould-shot, used interchangeably with an intermediate form of ‘horse shoe mould’ as evidenced by the eighteenth-century parish registers of Whitechapel. In Whitechapel’s burial register these descriptors exclusively represent infant burials. They are used for 63 infants dying in the years 1744–1747 whose age is between 1 day and 10 months, 10 of whom are less than one month old.^51^ These descriptors indicate abnormalities in the development of the skull bones: the normal shape of the fontanelle or ‘mould’ has been deformed. Several mid-seventeenth-century weekly Bills of Mortality use not head-mould-shot but mouldfallen as a further alternative descriptor that again accentuates a deviation from the expected bone shape.^52^

Glisson and Whistler referred to the deformities of the head in children with rickets under the general term hydrocephalus, also translated by Armin as ‘Dropsie in the Head’, and hydrocephalus as a term persisted in the medical literature and eventually crossed over to become a cause of death descriptor.^53^ But hydrocephalus is indicative of other childhood conditions besides rickets, including meningitis and spina bifida, for example.

In the late nineteenth century, clinically observed ‘rickets hydrocephalus’ could describe swellings of the head associated with rickets, and a new wave of autopsies revealed the brains of severely rachitic children to be large and ‘watery soft’.^54^ From the post-1750 burial register evidence, terminology for describing swollen heads passes through, in rough chronological sequence: dropsy on/in the brain/head; water in the brain/head; and finally hydrocephalus, and all of these could be implicated in rickets morbidity. The age incidence of these descriptors is much less tight than for horseshoe head and headmouldshot, although nearly all are children. Eighteenth century deaths attributed to hydrocephalus extend beyond London and are also found in other industrial areas, for instance in Leeds, Liverpool, York, and Newton Heath near Manchester. Since we have no burial registers with cause of death before 1750 except in London it is unfortunately not possible to say whether head-mould-shot and horseshoe head were also used more widely in the first half of the eighteenth century.

## Medical response: inspecting the dead and treating the living

On 27 January 1657/8, the diarist John Evelyn sorrowfully recorded the death of a much-beloved 5-year-old son at his house in Deptford near London. The intertwining of medical and lay involvement in sickness and death in a wealthy household is readily apparent. It was not customary for physicians to attend deathbeds, and children’s ailments were not their priority. Evelyn and his parents were attended by up to three physicians at a time for their own illnesses, but for his son’s illness we hear only of ‘the women and maids that attended him’, and no medical presence or treatment is mentioned, although after the child’s death Evelyn allowed a post-mortem to be performed, presumably by a physician.^55^ Evelyn at first states that the death occurred after a ‘quartan ague’ (recurrent fever) and then recounts the supposed cause of death not in medical terminology but as what is ‘vulgarly called liver-grown’, a descriptor used in the Bills of Mortality and one that his contemporary Graunt puzzled over. Graunt declared himself unable to ascertain the meaning of liver-grown, having considered but rejected it as a synonym for rickets on the basis of the statistical evidence.^56^

Rickets was initially noticed and reported by women. The first known manuscript rickets reference may have appeared as early as 1632, in a lady’s collection of home remedies.^57^ The first printed mention of rickets is in the 1634 London Bills of Mortality. The Bills were compiled from information provided by the two female Searchers of the Dead appointed in each parish. Searchers were chosen from among the respectable poor matrons of each of London’s 120 to 130 parishes. They were responsible for viewing the bodies of the deceased, and reporting the cause of death to the parish clerk. Despite their subsequently poor reputation in the historiography of the Bills of Mortality, they were respected contributors to parish governance.^58^ All of the statistical investigations that proceed from the Bills depend on the Searchers’ work, but their own direct testimony of identifying causes of death is not available to us. Their reports were most likely delivered orally, for female illiteracy was still the norm, even in London, and ability to write lagged some way behind ability to read. In the 1660s, only one London or Middlesex woman in five could sign her name in court depositions.^59^

The Searchers of the Dead had the opportunity to acquire broad and frequent experience of disease and death. Older women were the natural choice for the role because it was women who nursed the sick and laid out the dead for burial.^60^ Contemporaries perceived older women as seasoned against infectious disease partly from nursing children, a survival advantage that helped Searchers withstand regular close proximity to contagion long enough to gain further experience in office.^61^ Some Searchers remained in post for decades.^62^ High rates of mortality among large London populations gifted them with many bodies to observe. Large suburban parishes like St Giles Cripplegate generated upwards of 20 corpses every week, of which more than half could be expected to be children. During epidemics, the supply of corpses might rise fivefold or more.

Inclusion of rickets in the Bills of Mortality brought rickets to the attention of the literate, but knowledge of the name of a disease was not necessarily accompanied by awareness or consensus on what it meant. A sense of evolving medical perceptions of rickets can be gained from early printed books. Using published sermons and plays, it is also possible to gain a sense of what was popularly understood by the disease in the oral culture that the Searchers were part of, but space constraints mean this will not be fully addressed here.

Folk medicine practitioners of both sexes responded early to demand for a cure for rickets. A 1637 diatribe against quackery in London by Thomas Brian, MP excoriates ‘Donnington in Moore-fields, who […] burnes children behind the eares for the Rickets’.^63^ The same cure for ‘the new disease […] called the Ricketts’ was mentioned by the clergyman and popular preacher Thomas Fuller in a London edition of his homilies ten years later in 1647 (but not in the first edition published in Exeter in 1645). He writes of ‘a woman in the west who hath happily healed many [of rickets] by cauterizing the vein behind the ear’, and also develops a religious metaphor out of the symptoms of swollen head and shrunken limbs that would prove irresistible to subsequent sermonisers.^64^

Leading apothecaries were quick to publish rickets treatments and descriptions of its symptoms. The first apothecary to refer to rickets in print was John Parkinson, the King’s Herbalist, in his *Theatrum Botanicum* of 1640. He mentions rickets just once, following a repetition of Galenic theories on unlocking muscles or joints that have become immobile following spasms or convulsions.^65^ His description of rickets focuses on impaired growth, asserting that rickets ‘suffereth them [ie, children] not to grow or prosper eyther in height strength or alacritie’.

A more controversial apothecary, the College of Physicians’ adversary Nicholas Culpeper, provided a wide range of medicines suitable to relieve rickets in his *London Dispensatory* of 1649. This work is an embellished translation from the Latin of the College of Physician’s standard reference, the *Pharmacopeia Londinensis* of 1619. The original does not mention rickets, but Culpeper’s version contains some 20 references to medicines that might alleviate the disease, giving an indication of growing demand for rickets cures. Among these is an acknowledgement that the name and identity of the disease comes from women’s knowledge of childhood maladies, for Culpeper refers to: ‘that disease in children which women call the Rickets’.^66^ Thomas Sherwood, a self-proclaimed London ‘practitioner in physic’ had recommended a cure for ‘the Rickets in children’ in 1641 that linked rickets with liver conditions, in an optimistically comprehensive short work whose title boasted of cures for both plague and smallpox.^67^ Culpeper takes the link to liver conditions one step further to equate rickets with the mysterious condition ‘livergrown’ later rejected by Graunt as a synonym for rickets in the Bills of Mortality. Culpeper’s rickets treatments include remedies for toothache and for relieving swellings of the hands, feet, knees and joints.^68^

None of the early rickets remedies known to us could have cured Vitamin D deficiency. The best prospect recent scholars have identified are crows’ livers, an admittedly unlikely source of Vitamin D.^69^ Cod liver oil, which contains high concentrations of Vitamin D, was not known medically until the late eighteenth century. In 1782 it was successfully administered orally to elderly patients at the Manchester Infirmary with ‘rheumatism’ (possibly osteomalacia), although it had long been reputed popularly as a rickets remedy in Holland, and cod livers were part of the diet of northern Scotland.^70^ Calcium-rich foods were rarely mentioned in rickets remedies, although one printed in the 1650s advocated regular drinking of well-boiled milk. Boiling would have prevented bacterial infection but also destroyed Vitamin C that might otherwise have helped alleviate scurvy, if also present. Other remedies were applied externally: cream or butter featured in another, but only for use in a massage oil.^71^

By the mid-1640s, medical treatises on rickets began to emerge. In 1645, 1649 and 1650 physicians published the first comprehensive descriptions of rickets symptoms, the earliest by Daniel Whistler, born in Walthamstow near London and an Oxford medical student who completed his education at Leiden; the second a chapter within an untranslated book by Arnold Boate, a Dutch physician also trained at Leiden who practised in London and Dublin (his brother Gerard Boate’s natural history of Ireland published in 1652 also noted the recent appearance of rickets there); and the last the best-known, by Cambridge-trained physician Francis Glisson, whose treatise was the result of a London College of Physicians enquiry on the subject.^72^ Both Glisson and Whistler provided comprehensive itemised descriptions of rickets symptoms and speculated on the disease’s origins. These descriptions give the clinical features of rickets in detail, and Glisson’s treatise continues to be accepted as the authoritative early medical work on rickets. We may be confident that they were observing rickets as understood in modern medicine.

Rickets was not absent from medical notice in the eighteenth century, even as it disappeared from the London Bills of Mortality as a cause of death. More than forty theses entitled ‘De Rachitide’ were presented between 1704 and 1799 at universities throughout northern and central Europe, and especially at Edinburgh, which contributed eight between 1731 and 1787, more than any other institution.^73^ In 1706 the physician John Floyer, who had practised in the town of Lichfield in the Midlands since 1675, described rickets as the most prevalent infant disease.^74^ Its continuing presence in London half a century later in 1753 is confirmed by Nelson, who reported it to be ‘extremely common’.^75^ However, in 1767, the physician George Armstrong evinced a markedly changed attitude towards the risk represented by rickets when he declared that rickets was not to be accounted a fatal condition, and also gestured towards its geographical specificity, only having encountered cases among nurse children sent from London.^76^ But the disease continued to be seen as an English problem by outsiders. A Spanish visitor, Don Gonzales, who returned home in 1730, counted rickets first among three diseases to which the English were peculiarly susceptible, the others being scurvy and consumption.^77^ William Fordyce, writing in 1773, described the swelled wrists and ankles or crooked limbs he reckoned to be suffered by 20,000 children in London, the large numbers arising because he accounted the disease particularly prevalent among the poor. However, he did not call the malady he observed rickets, but rather a hectic fever, as suited the subject of his treatise.^78^

Whistler’s and Glisson’s medical treatises on rickets both refer to autopsies conducted on rickets sufferers, although neither says how many bodies were examined. Their most likely source of corpses with known clinical histories were the well-off patients they themselves, or their acquaintances, treated, when consent was forthcoming from parents, as from Evelyn for his liver-grown child.^79^ Both discuss the ages at which children are generally affected by the disease, and it is illuminating to consider this in light of the epidemiological evidence discussed above in Section I.

Whistler declares that rickets is a disease of infants, a term then usually meaning children under 2 years of age, with onset between the first year of life and the fourth. Neonates are within his purview, for he declares that rickets is invariably fatal to children suffering from rickets at or before birth. He refers to rickety children being unable to stand, which in the modern world is expected of a normally developing child between 7 and 17 months old, according to the World Health Organisation’s motor milestones.^80^ Cases in children who have already begun walking, which would mean by 18 months by today’s standards, and in children still older, he says are more curable, and less mortal.^81^ Glisson gives more specific age information, and for him too rickets is, by titular definition, a disease of young children. He asserts that rickets affects children aged over 6 months and under 5 years, and predominantly those aged between 9 and 30 months, with peak incidence above 18 months.^82^ He knows of only one case of neonatal rickets, but states that the sooner after birth a child is affected, the more likely it is to die.^83^

Whistler and Glisson also make some observations on the social status of those affected by rickets, in giving their opinions on its dietary causes. Whistler understood rickets to be a disease of the rich and the poor, but less of the middling sort who he believed to be more moderate in diet. Glisson concerns himself almost entirely with the wealthy, among whom he thought rickets most prevalent. Whistler and Glisson’s remarks are particularly noteworthy for what they tell us about seventeenth-century infant and child feeding practices.

Glisson discusses infant feeding practices in the context of why children are susceptible to rickets at particular ages, declaring that breast-milk is the best food for newborn infants and that, by providing perfect nutrition, it protects them from rickets, all the while hinting that this was not a typical infant diet.^84^ Elsewhere he implicates over-feeding as a cause of rickets insofar as it explains why ‘the Cradles of the rich’ are afflicted more with rickets than those of the poor.^85^ On humoural principles, for those prone to rickets he deprecates fresh and salted fish, fresh meat and meat that has not been ‘concocted’ (turned into soup), salted or smoked meat, spiced meat, all cheeses, newly baked bread, and sweetened food unless mixed with wine; advocating that those suffering with rickets should instead adopt a ‘thin spare diet’.^86^ Whistler directly lays the blame for rickets on improper feeding of children at early ages. He asserts that English children are unlike any others in the world in being fed large quantities of meat from a very young age.^87^ He also condemns the practice of hiring a wetnurse on the basis that mothers’ milk, and especially the colostrum, is a more suitable food for young infants (had Whistler published in English, he would have been the earliest known advocate of the colostrum in the vernacular medical literature, rather than the German physician Ettmueller, in translation).^88^ Whistler’s response to a question appended to his thesis affirms his support for maternal breastfeeding.^89^ Nonetheless, like Glisson, Whistler does not propose milk of any kind as a remedy for rickets, despite intuiting its protective effects in infants.

Glisson takes it for granted that childcare is provided by nurses, as it would have been among his patients. He makes no mention of mothers’ involvement in any hands-on aspect of childcare: feeding, clothing and exercising infants are all portrayed as the nurse’s responsibility. The competence of nurses is questioned frequently, as when, for example, children are said to be over-clothed or swaddled (which would have reduced the amount of sunlight reaching the skin). The usual childcare method among the wealthy of early modern London was to hire a wetnurse, initially by sending the child away to the nurse’s home, although by the second half of the seventeenth century, nurses resident in the mother’s home were growing more common.^90^ Despite his previous remarks on unsuitable foods for rickety infants, Glisson seems to assume that ‘Breast-milk or other Milk’ is what nurses generally feed their charges, to the age of nine months.^91^ His stress on the further benefit to the infant of suckling the milk direct from the breast admits the existence of other possibilities, such as the use of pap-boats or feeding bottles, associated with feeding substances other than breast-milk.^92^ Glisson also asserts that weaning may introduce ‘dietary errors’.^93^ Despite this, he makes no suggestion that continuing to provide milk might alleviate rickets symptoms, and advocates early weaning if the nurse has bad milk, is pregnant, over-fond of sex, sick, drunk or gluttonous; advice that further disparages nurses and suggests that availability of suitable candidates could not be assumed.

Nurses did not always feed their charges breast-milk, or indeed any kind of milk. Mrs Wright’s eighteenth century medical advice is illuminating on the continuing perceived superiority of meat. As she explained: ‘It sometimes happens, that wet nurses have not enough of milk for their nurselings, and make up their food with bread, tapioca, or Indian arrow root, and the common milk of this town; we believe, that in general, beef tea, mutton or veal broth, mixed with them, is preferable’.^94^ Milk was not considered essential in children’s diets, and was in any case not always available. There is some evidence for increasing consumption of dairy in the eighteenth century, and cheese was provided in London workhouses in the 1710s and 1720s, but for the most part there is little information on what foods reached the mouths of children.^95^ In 1757 the London Foundling Hospital, which tried to shape its feeding practices to the best observed child mortality outcomes and thus wetnursed many of its infants, counting prominent physician advocates of breast-milk like Hans Sloane among its governors, used a pap of grated flour-and-water biscuit softened with water mixed with goat’s or cow’s milk for those of its infants who were dry nursed, but after 1759 milk was omitted even there.^96^ Older children at the Foundling Hospital were given small beer, after a period of experimentation with water mixed with milk instead.^97^ The benefits of consuming animal milk in averting calcium deficiency and rickets must have been offset by the dangers of bacterial infection. Outside London, there were considerable regional differences in the domestic availability of milk, with just one pint per week consumed in a well-off Berkshire household in 1744 compared to 51 pints per week in a poor Cumberland household.^98^ Longstanding regional differences in milk production may help to explain why rickets in rural areas was better known in the south of England than in the North. In the mid-seventeenth century, Glisson asserted the presence of the rickets in Dorset, Somerset and throughout western and southern England, but less in the North, whereas Whistler includes an etymology for the word rickets from a Dorset dialect word.^99^ More than two centuries later, as noted above, the British Medical Association geographical survey of 1889 also showed rural rickets to be more of a problem in southern England.

## Conclusion

In the seventeenth century, there was an accord between increased medical interest and surveillance of rickets and statistical evidence of its presence, but this unusual consensus fell apart when the disease no longer seemed lethal to contemporaries. Rickets was not subsequently used in the nineteenth century as a certified cause of death, and in that period it has been described as a hidden disease precisely because despite its high incidence it was not regarded as lethal, attracting little public health concern, a reversal of the seventeenth-century reaction to the disease.^100^ This diagnostic paradox invites consideration of the wider motivation for stating that a particular disease is a cause of death.

An important consideration in why particular cause of death descriptors are used is the balance between newness and familiarity, and the contextual knowledge of the reporter. A childhood disease must have needed to be common enough and distinctive enough for carers to see the same symptoms repeatedly before it was named and adopted by the Searchers as a cause of death. Conversely, symptoms that are very common eventually cease to seem noteworthy unless their dangers are immediate (as with the smallpox rash, for example). When a disease becomes ubiquitous, we may have trouble perceiving its outward manifestations at all. The present global obesity epidemic offers an example of this phenomenon.^101^ From visual inspection an overweight child is now frequently judged normal, by peers, parents and even by doctors, while normal children may be perceived as underweight.^102^ Similarly, rachitic symptoms may have occurred so frequently among young children in eighteenth century London that rickets became unremarkable and could no longer be considered a plausible cause of death, especially as it became evident that many young children survived the disease. In Glisson’s account of 1650, there is already explicit acknowledgement that the condition may ameliorate ‘by the sole benefit of Age[ing]’.^103^

The circumstances of the seventeenth-century fatal rickets epidemic that was apparently peculiar to England, and London in particular, also demand explanation, and this has been the main motivation for this article. There were sufficient fatalities of this apparently new disease among the children of wealthier citizens to induce a medical enquiry, and evidence of pre-nineteenth century rickets among Londoners of all social classes survives in statistical records, osteological remains and contemporary medical observations.

This early rickets epidemic attests to the fragility of infant and child health in response to adverse conditions in England that were in this case largely anthropogenic, tipping the precarious climatic availability of Vitamin D at high latitudes into unavailability through urban air pollution from the new dependence on coal as a fuel in the growing metropolis, not yet accompanied by the technology to burn it more cleanly. This was exacerbated by deficient infant diets. Rickets-induced hypocalcaemia, convulsions, or additional nutritional deficiencies causing scurvy, could genuinely be lethal. Other child deaths attributed to rickets were likely recorded by the Searchers as such because of attention-grabbing deformities, especially to the head, while increased susceptibility to infectious diseases, and respiratory disease in particular, were more probable true causes of death. In the eighteenth century as well as the nineteenth, rickets morbidity almost certainly contributed to whooping cough mortality.

In the section ‘The epidemiological evidence’ we saw that a much higher proportion of rickets deaths could occur from rickets in a rich parish like St Michael Cornhill than a poor one like St Giles Cripplegate, and contemporary medical authorities affirm that rickets was particularly prevalent among the wealthy. Two aspects to the unequal experience of rich and poor children with rickets have been identified. Firstly, given the age incidence of rickets in children of differing status confirmed by osteological evidence, rickets affected rich young infants earlier and more severely. Differences in feeding regimen by social status supply the most likely reason for nutritional deficiencies of calcium and perhaps Vitamin C, over and above deficiencies in Vitamin D induced by smog-related lack of sunlight experienced by all Londoners. Unlike poorer women, high status women did not usually breastfeed their own infants, and in consequence their infants were less likely to be fed exclusively on breast-milk. Secondly, where rich children survived, their recovery was better, so that few past the age of 2 years could plausibly be thought to die of the disease even when rickets was thought more lethal. Nutritional status may be implicated in this difference too. Richer families could afford to set their tables with more varied produce, so those of their children who survived weaning could access a wider range of nutrients, promoting faster recovery. Another reason for the difference in recovery of high status children may relate to their chances of escaping the city smog and experiencing sunlight outside London, at least periodically, on recreational excursions outside the city, while visiting family members, or staying in countryside residences. While in London, children who lived with richer families were also more likely to live on the wider main streets rather than densely packed and shaded courts or alleys. A limiting factor for outdoor pursuits of all children who suffered from rickets in early childhood was the difficulty and delay in walking unsupported. But for the well-off, there were servants or nursemaids to carry them about, supportive clothing, and even go-carts, mechanical contraptions devised to aid stumbling steps. Such recourse would not have been available to poor children, who were more likely to remain housebound until they were able to walk unaided.

By weakening resilience, environment and nutrition contribute to morbidity and mortality above and beyond the number of fatalities that can be observed directly. Just as rickets seems to have paved the way for the emergence and rise of whooping cough in eighteenth century London, environmental and nutritional factors more generally have an impact on the incidence and lethality of infectious disease, then responsible for the majority of deaths, especially in cities. Much of what we now know of seventeenth-century rickets stems from a concern for the health outcomes of wealthy children, for whom micronutrient deficient infant feeding practices were probably the most significant casual factor, given the likelihood of opportunities to escape urban air pollution, at least periodically. Scrutiny of infant and childhood diets and recognition of their importance to health, as evinced in seventeenth-century physicians’ investigations of rickets, albeit as yet unaccompanied by clear advice or consensus on what constituted a good diet, has far-reaching implications. Through gradual shifts in attitude to child feeding and awareness of its importance, there was the potential for the well-off to overtake the rest in morbidity outcomes, leading to new social gradients in mortality yet to come.

## Funding

This work was supported by the Wellcome Trust, reference 360G-Wellcome-103322_Z_13_Z

## Footnotes

1 George Alter and Ann Carmichael, ‘Studying Causes of Death in the Past: Problems and Models’, *Historical Methods*, 1996, 29.

2 George C. Alter and Ann Carmichael, ‘Classifying the Dead: Toward a History of the Registration of Causes of Death’, *Journal of the History of Medicine and Allied Sciences*, 1999, 54, 120–22. A notable exception is Graunt’s friend the polymath William Petty, who studied medicine at Oxford and later proposed reformatting the Dublin Bills of Mortality.

3 See E. A. Wrigley *et al.*, *English Population History from Family Reconstitution 1580-1837* (Cambridge: Cambridge University Press, 1997), 248–63; John Landers, *Death and the Metropolis: Studies in the Demographic History of London 1670-1830* (Cambridge: Cambridge University Press, 1993), 134–36.

4 G. T. Smerdon, ‘Daniel Whistler and the English Disease: A Translation and Biographical Note’, *Journal of the History of Medicine and Allied Sciences*, 1950, 5, 397–415; Suma Uday and Wolfgang Högler, ‘Prevention of Rickets and Osteomalacia in the UK: Political Action Overdue’, *Archives of Disease in Childhood*, 2018, 103, 901–906.

5 Scientific Advisory Committee on Nutrition*, Vitamin D and Health* (2016), Crown Copyright, https://www.gov.uk/government/publications/sacn-vitamin-d-and-health-report (last accessed February 2021).

6 Felix Bronner and Danielle Pansu, ‘Nutritional Aspects of Calcium Absorption’, *Journal of Nutrition*, 1999, 129, 9–12.

7 Michael F. Hollick, ‘Environmental Factors that Influence the Cutaneous Production of Vitamin D’, *The American Journal of Clinical Nutrition*, 1995, 61, 638S–645S, 641S–642, 638S.

8 Anne Hardy, ‘Bread and Alum, Syphilis and Sunlight: Rickets in the Nineteenth Century’, *International Journal of Epidemiology*, 2003, 337–40, 340.

9 Bill Luckin and Peter Thorsheim, eds., *A Mighty Capital under Threat: The Environmental History of London, 1800-2000* (Pittsburgh: University of Pittsburgh Press, 2020), 10, 70–72, 105, 184.

10 Peter Brimblecombe, *The Big Smoke: A History of Air Pollution in London since Medieval Times* (London and New York: Methuen, 1987, repr. Routledge, 2011), 54.

11 John Evelyn, *Fumifugium, or, the Inconveniencie of the Aer and Smoak of London Dissipated […]* (London, printed by W. Godbid for G. Bedel and T. Collins, 1661), 6–7.

12 William M. Cavert, *The Smoke of London: Energy and Environment in the Early Modern City* (Cambridge: Cambridge University Press, 2016), 84–98.

13 Brimblecombe, *The Big Smoke*, 46.

14 John Hatcher, *History of the British Coal Industry Volume 1: Before 1700: Towards the Age of Coal* (Oxford: Oxford University Press, 1993), 501.

15 Phil Jones, ‘Historical Climatology: A State of the Art Review’, *Weather*, 2008, 63, 181–186, Figure 2.

16 R. W. Unger, ‘Energy Sources for the Dutch Golden Age: Peat, Wind, and Coal’, *Research in Economic History*, 1984, 9, 221–53.

17 Eunhye Kim and Sabine Barles, ‘The Energy Consumption of Paris and its Supply Areas from the Eighteenth Century to the Present’, *Regional Environmental Change*, 2012, 12, 295–310, 300.

18 Paul Warde, ‘Energy and Natural Resource Dependency in Europe 1600-1900’, Brooks World Poverty Institute Working Paper 77, University of Manchester, 2009, Figure 2, n.p.

19 Ben Coates, *The Impact of the English Civil War on the Economy of London, 1642-50* (Aldershot: Ashgate, 2004, repr. London: Routledge, 2014), 146.

20 Vanessa Harding, ‘Families and Housing in Seventeenth Century London’, *Parergon*, 2007, 24, 115–38.

21 William Baer, ‘Housing for the Lesser Sort in Stuart London: Findings from Certificates, and Returns of Divided Houses’, *The London Journal*, 2008, 33, 61–88.

22 Causes of Death Burials Dataset and Family History Society Burials Dataset, Cambridge Group for the History of Population and Social Structure; see https://doi.org/10.17863/CAM.40869 (last accessed February 2021).

23 John Haygarth, ‘Observations on the Bill of Mortality, in Chester, for the Year 1772, By Doctor Haygarth’, *Philosophical Transactions*, 1774, 64, 67–78, Table II.

24 Exact calendar months cannot be aggregated from the weekly bills. Rather than splitting weekly totals to derive calendar months, a method closer to the original data has been pursued here. The 13 consecutive periods are each 28 days long and comprise four weekly bills. Period 1 begins on dates varying between 12 and 21 December (in the Julian calendar, then running 10–11 days behind the present Gregorian calendar).

25 Widows and pensioners are among those dying of scurvy in St Giles Cripplegate, London in the 1670s, for example. London Metropolitan Archives, Parish Registers of St Giles Cripplegate 1672-1679, P69/GIS/A/002/MS06419/008.

26 Franics Glisson, *A Treatise of the Rickets being a Diseas Common to Children […], Translated into English by Phil. Armin* (London, printed by Peter Cole, 1651), 227.

27 Philip R. Evans, ‘Infantile Scurvy: the Centenary of Barlow's Disease’, *British Medical Journal*, 1983, 287, 1862–63.

28 London Metropolitan Archives, St Michael Cornhill Parish Register 1653–1736, P69/MIC2/A/003/MS04063, Item 001.

29 9 were linked directly to a baptism record. The age of the tenth could be set within bounds through the prior baptism of a sister described as the eldest. Record linkage in London’s high population turnover environment is usually disproportionately successful among those who die young. But here since a high proportion of all rickets burials were traced that any bias will be negligible. The interval between birth and baptism was short: in four instances where it is known it was 9–16 days, and presence of a birth date is not selective but a consequence of the baptism register changing format.

30 ‘Hearth Tax: City of London 1662, Cornhill ward’, in Centre for Metropolitan History, *London Hearth Tax: City of London, 1662*, 2011, http://www.british-history.ac.uk/london-hearth-tax/london/1662/cornhill-ward (last accessed February 2021).

31 Thomas R. Forbes, ‘Sextons’ Day Books for 1695-7 and 1694-1703 from the parish of St Martin-in-the-Fields, London’, *Yale Journal of Biology and Medicine*, 1973, 46, 142–50.

32
*Ibid*., 146.

33 London Metropolitan Archives, St Giles Cripplegate Parish Register 1653–57, P69/GIS/A/002/MS06419/005.

34 Vivian Brodsky Elliott, ‘Mobility and Marriage in Pre-Industrial England*’* (unpublished PhD thesis, Cambridge University, 1978), 81, 115.

35 London Metropolitan Archives, St Helens Bishopsgate Composite Parish Register 1649–95, P69/HEL/A/002/MS06831.

36 London Metropolitan Archives, St Stephen Coleman Street Composite Parish Register 1636–1717, P69/STE1/A/002/MS04449.

37 London Metropolitan Archives, St John Wapping Register of Burials 1665–1707, P93/JN2/023.

38 Transcribed eighteenth century account books for St Dunstan Stepney 1733–1843 and St Martin-in-the-Fields 1747–1825 were also searched for rickets casualties, but only one found.

39 Landers, *Death and the Metropolis*, 136–38; Gill Newton and Richard Smith, ‘Convergence or Divergence? Mortality in London, its Suburbs and its Hinterland between 1550 and 1700’, *Annales de Démograhie Historique*, 2013, 2, 17–49, 28–29.

40 Gill Newton, ‘Infant Mortality Variations, Feeding Practices and Social Status in London between 1550 and 1750’, *Social History of Medicine*, 2011, 24, 260–80, 275–77; 271–73.

41 Romola Davenport, ‘Infant-feeding Practices and Infant Survival by Familial Wealth in London, 1752–1812', *The History of the Family*, 2019, 24, 174–206, 189–90.

42 S. Mays, ‘The Rise and Fall of Rickets in England’, in P. Murphy and P. E. J. Wiltshire, eds., *The Environmental Archaeology of Industry* (Oxford: Oxbow, 2003), 144–53.

43 Theya Molleson *et al.*, *The Spitalfields Project Volume 2 The Anthropology: The Middling Sort* (Council for British Archaeology Research Report 86, 1993).

44 R. Pinhasi *et al.*, ‘Morbidity, Rickets and Long-bone Growth in Post-medieval Britain—a Cross-population Analysis’, *Annals of Human Biology*, 2006, 33, 372–89, 375.

45
*Ibid*., 373, 376.

46 John S. Parry, ‘Observations on the Frequency and Symptoms of Rachitis, with the Results of the Author's Clinical Experience’, *The American Journal of the Medical Sciences*, 1872, 63, 17–52, 17.

47 Parry, *Rachitis*, 48–49.

48 Lulu Muhe *et al.*, ‘Case-control Study of the Role of Nutritional Rickets in the Risk of Developing Pneumonia in Ethiopian Children’, *The Lancet*, 1997, 349, 1801–04; Abdelhamid S. Najada, Moen S. Habashneh and Maher Khader, ‘The Frequency of Nutritional Rickets among Hospitalized Infants and its Relation to Respiratory Diseases’, *Journal of Tropical Pediatrics*, 2004, 50, 364–68.

49 Anne Hardy, ‘Rickets and the Rest: Childcare, Diet and the Infectious Children’s Diseases 1850-1914’, *Social History of Medicine*, 1992, 5, 389–412.

50 Charles Creighton, *A History of Epidemics in Britain Volume II: From the Extinction of Plague to the Present Time* (Cambridge, Cambridge University Press, 1894), 655, 669.

51 London Metropolitan Archive, St Mary Whitechapel Burials Register 1743–1767, P93/MRY1/062.

52 Weekly London Bills of Mortality for 1664-5 weeks 9, 12 and 18.

53 Glisson, *Rickets*, 247; Smerdon, ‘Whistler: a Translation’, 403, 406.

54 Parry, ‘Rachitis’, 44.

55 William Bray, ed., *The Diary of John Evelyn: Volume 1*, (Walter Dunne, Washington and London, 1901), 320–22.

56 John Graunt, *Natural and political observations made upon the Bills of Mortality* (London, printed by T. Roycroft for J. Martin, J. Allestry and T. Dicas, 1662), 25.

57 Layinka M. Swinburne, ‘Rickets and the Fairfax Family Receipt Books’, *Journal of the Royal Society of Medicine*, 2006, 99, 391–95.

58 Richelle Munkhoff, ‘Searchers of the Dead: Authority, Marginality and the Interpretation of Plague in England 1574-1665’, *Gender and History*, 1999, 11, 1–29.

59 David Cressy, *Literacy and the Social Order: Reading and Writing in Tudor & Stuart England* (Cambridge: Cambridge University Press, 1980), 142–47.

60 Deborah E. Harkness, ‘A View from the Streets: Women and Medical Work in Elizabethan London’, *Bulletin of the History of Medicine*, 2008, 82, 52–85. Richelle Munkhoff, ‘Poor Women and Parish Public health in Sixteenth-century London’, *Renaissance Studies*, 2014, 28, 579–96.

61 Margaret Pelling, ‘Skirting the city? Disease, Social Change and Divided Households in the Seventeenth Century’, in Paul Griffiths and Mark S. R. Jenner (eds.), *Londinopolis: Essays in the Cultural and Social History of Early Modern London* (Manchester and New York, Manchester University Press, 2000), 154–75, 169.

62 Munkhoff, ‘Searchers’, 18–19.

63 Thomas Brian, *The Pisse-prophet […]* (London, printed by E.P. for R. Thrale, 1637), 103–04.

64 Thomas Fuller, *Good Thoughts in Worse Times* (London, printed by W. W. for J. Williams, 1647), 141–42.

65 John Parkinson, *Theatrum botanicum […]* (London, printed by Thomas Coates,1640), 980.

66 Nicholas Culpeper, *A Physical Directory, or, A Translation of the London Dispensatory made by the Colledge of Physicians in London* […] (London, printed for Peter Cole, 1649), 129.

67 Thomas Sherwood, *The Charitable Pestmaster* […] (London, printed by A.N. for John Francklin, 1641), 15.

68 Culpeper, *Physical Directory*, 129.

69 Jeffrey O. H. O’Riordan, ‘Rickets in the 17^th^ Century’, *Journal of Bone and Mineral Research*, 2006, 21, 1506–10, 1509.

70 Ruth A. Guy, ‘The History of Cod Liver Oil as a Remedy’, *American Journal of Diseases of Children*, 1923, 26, 112–16.

71 For example in A.M., *Queen Elizabeth’s closset of physical secrets* […] (London printed for William Sheares Junior, 1656), 63; Anon, *The Queen’s closet opened*, (London, printed for Nathaniel Brooke, 1659), 126.

72 Daniel Whistler, *Disputatio […] de morbo puerili Anglorum, quem patrio idiomate indigenae vocant The Rickets […]* (Leiden, printed by Wilhelm Christian Boxius, 1645); Arnold Boate, *Observationes Medicae de Affectibus Omissis* […], (London, printed by Tho. Newcomb for Tho. Whitaker, 1649); Francis Glisson, *De rachitide, sive, Morbo puerili qui vulgo The Rickets dicitur, tractatus opera primo ac potissimum Francisci Glissonii […] adscitis in operis societatem Georgio Bate & Ahasuero Regemorto […]*, (London: printed by Guil. Du-gardi for Laurentii Sadler & Roberti Beaumont, 1650).

73 Wilhelm G. de Ploucquet, *Literatura Medica Digesta Volume IV*, (Tubingen, 1809), 11–12.

74 Denis Gibbs, ‘Rickets and the Crippled Child: an Historical Perspective’, *Journal of the Royal Society of Medicine*, 1994, 87, 729–32, 730.

75 J. Nelson, *An Essay on the Government of Children […]*, (London, 2nd edn., printed for R. and J. Dodsley, 1756), 96.

76 George Armstrong, *An Essay on the Diseases most Fatal to Infants* (London, 1767), 96.

77 M. Don Gonzales, *A Collection of Voyages and Travels*, (London, 1747),197.

78 William Fordyce, *A New Inquiry into the Causes, Symptoms and Cure of Putrid and Inflammatory Fevers: with an Appendix on the Hectic Fever and on the Ulcerated and Malignant Sore Throat* (London, 1773), 207.

79 David Harley, ‘Political Post-mortems and Morbid Anatomy in Seventeenth-century England’, *Social History of Medicine*, 1994, 7, 1–28, 3.

80 Smerdon, ‘Whistler: a Translation’, 408–11.

81
*Ibid*., 411.

82 Glisson trans. Armin, *Rickets*, 192–93.

83
*Ibid*., 154.

84
*Ibid*., 194.

85
*Ibid*., 170.

86
*Ibid*., 169.

87 Smerdon, ‘Whistler: a Translation’, 408.

88 Fildes, *Breasts, Bottles and Babies*, 86.

89 Smerdon, ‘Whistler: a Translation’, 408, 415.

90 Newton, ‘Infant Mortality Variations’, 273–78.

91 Glisson trans. Armin, *Rickets*, 195.

92 Valerie Fildes, *Breasts, Bottles and Babies: a History of Infant Feeding* (Edinburgh, Edinburgh University Press, 1986), 343–50.

93 Glisson trans. Armin, *Rickets*, 196.

94 Mrs Wright, *An Essay to Instruct Women How to Protect Themselves in a State of Pregnancy […]* (London, J. Barker, Lee and Hurst and J. Kirby, 1798), 26.

95 Craig Muldrew, *Food, Energy and the Creation of Industriousness* (Cambridge: Cambridge University Press, 2011), 116, 134.

96 Alysa Levene, *Health and Survival Chances at the London Foundling Hospital and the Spedale Degli Innocenti of Florence, 1741–*99, (unpublished PhD thesis, Cambridge University, 2002), 166.

97
*Ibid*., 78.

98 Muldrew, *Food*, 137–39.

99 Glisson trans. Armin, *Rickets*, 4; Smerdon, ‘Whistler: a Translation’, 401.

100 Hardy, ‘Bread and Alum’, 337.

101 K. Maximova *et al.*, ‘Do You See What I See? Weight Status Misperception and Exposure to Obesity among Children and Adolescents’, *International Journal of Obesity*, 2008, 32, 1008–15; R. Chaimovitz *et al.*, ‘Body Perception: Do Parents, their Children, and their Children's Physicians Perceive Body Image Differently? *Journal of Pediatric Gastroenterology and Nutrition*, 2008, 47, 76–80.

102 Jessica Doolen, Patricia T. Alpert and Sally K. Miller, ‘Parental Disconnect Between Perceived and Actual Weight Status of Children: A Metasynthesis of the Current Research’, *Journal of the American Association of Nurse Practitioners*, 2009, 21, 160–6.

103 Glisson trans. Armin, *Rickets*, 251.

## Notes

Gill Newton is a Research Associate at the Cambridge Group for the History of Population and Social Structure. She has wide interests in data-driven research in English population history, particularly focussed on urban areas. She has published on London’s infant and child mortality regime previously in *Social History of Medicine.* Recently she co-authored a book on entrepreneurs in the Victorian Censuses. She has also worked on Britain’s occupational structure.

